# Novel compound heterozygous *SUCLG1* variants may contribute to mitochondria DNA depletion syndrome‐9

**DOI:** 10.1002/mgg3.2010

**Published:** 2022-06-28

**Authors:** Yi‐ming Chen, Wei Chen, Yue Xu, Chao‐sheng Lu, Mian‐mian Zhu, Rong‐yue Sun, Yihong Wang, Yuan Chen, Jiaming Shi, Dan Wang

**Affiliations:** ^1^ Department of Surgery The Second Affiliated Hospital and Yuying Children's Hospital of Wenzhou Medical University Wenzhou Zhejiang China; ^2^ Department of Radiology The Second Affiliated Hospital and Yuying Children's Hospital of Wenzhou Medical University Wenzhou Zhejiang China; ^3^ Department of Pediatrics The First Affiliated Hospital of Wenzhou Medical University Wenzhou Zhejiang China

**Keywords:** compound heterozygous variants, mitochondrial DNA depletion syndrome 9, mitochondrial encephlomyopathy, *SUCLG1*, whole exome sequencing

## Abstract

**Background:**

Succinate‐CoA ligase/synthetase (SCS) deficiency is responsible for encephalomyopathy with mitochondrial DNA depletion and mild methylmalonic aciduria. Variants in *SUCLG1*, the nuclear gene encoding the alpha subunit of the SCS enzyme playing a pivotal role in maintaining mtDNA integrity and stability, are associated with mitochondrial DNA depletion syndrome 9 (MTDPS9).

**Methods:**

In this study, we reported an infant with clinical features of MTDPS9 from China. Whole exome sequencing (WES) was used to identify the genetic cause. Bioinformatic analysis and mtDNA level detection were performed to assess pathogenicity.

**Results:**

The proband manifested with hypotonia, lactic acidosis, mild methylmalonic aciduria, hearing loss and psychomotor retardation. WES identified new compound heterozygous *SUCLG1* variants of c.601A>G (p.R201G) in exon 6 and c.871G>C (p.A291P) in exon 8. Computational analysis predicted that these missense variants might alter structure stability and mitochondrial translocation of SUCLG1. qRT‐PCR showed 68% depletion of mtDNA content in proband as compared to controls.

**Conclusion:**

Novel compound heterozygous variants c.601A>G (p.R201G) and c.871G>C (p.A291P) in *SUCLG1* may cause MTDPS9 in this family. Our finding should be helpful for molecular diagnosis, genetic counseling and clinical management of SCS deficiency disorders.

## Graphical abstract

Novel compound heterozygous variants c.601A>G ( p.R201G) and c.871G>C (p.A291P) in SUCLG1 may be responsible for mitochondrial DNA depletion syndrome 9. Our finding should be helpful for molecular diagnosis, genetic counseling and clinical management of SCS deficiency disorder.

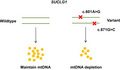

## 1 INTRODUCTION

Mitochondrial DNA (mtDNA) depletion syndromes (MDS) are genetically and clinically varied spectrum of autosomal recessive disorders featuring compromised mtDNA copy number in tissues and organs highly dependent on ATP production by oxidative phosphorylation, including brain, liver, muscle and heart (Viscomi and Zeviani, 2017; Stenton and Prokisch, 2020). Adequate content of mtDNA is essential for mitochondrial respiratory chain reaction and subsequent energy biosynthesis. To date, errors in 25 nuclear genes have been identified as causes of MDS. These genes are involved in the mitochondrial deoxynucleotide triphosphate (dNTP) supply for mtDNA nucleotides metabolism (*SUCLG1*, *SUCLA2*, *RRM2B*, *TK2*, *DGUOK*, *TYMP*, *ABAT*, *SLC25A4*, *AGK*, *MPV17*, *DTYMK*), mtDNA replisome (*POLG*, *POLG2*, *TWNK*, *DNA2*, *MGME1*, *TFAM*, *RNASH1*), mitochondrial communications (*OPA1*, *OPA3*, *MFN2*, *FBXL4*), protein folding and degradation (*GFER*, *SPG7*, *AFG3L2*) or other roles in mitochondrial maintenance (*MRM2*, *SLC25A21*, *SLC25A10*) (El‐Hattab and Scaglia, 2013; Garone et al., 2017; Viscomi and Zeviani, 2017; Molaei et al., 2020; Wang et al., 2020; Bychkov et al., 2021).

Succinyl‐CoA ligase/synthetase (SCS) is an enzyme in mitochondrial tricarboxylic acid cycle that reversibly catalyzes succinyl‐CoA to succinate and free CoA, and converts ADP or guanosine diphosphate (GDP) to ATP or guanosine triphosphate (GTP), respectively (Van Hove et al., 2010). In eukaryotes, SCS exists as a heterodimer composed of two subunits, one α subunit encoded by *SUCLG1* (OMIM: 611224) and one β subunit encoded by *SUCLA2* or *SUCLG2* (Johnson et al., 1998). Mitochondrial DNA depletion syndrome 9 (MTDPS9) is caused by biallelic *SUCLG1* variants. Patients with *SUCLG1*‐related MDS is characterized by encephalomyopathy with mtDNA depletion (Van Hove et al., 2010; Liu et al., 2016; Molaei et al., 2020). Symptoms include pronounced developmental delay/cognitive impairment, growth retardation, feeding difficulty, failure to thrive, hepatopathy, sensorineural hearing loss, and dystonia. Biochemical findings are mild methylmalonic aciduria and severe lactic acidosis (Liu et al., 2016). Life expectancy is usually short, with median survival of 20 months (Carrozzo et al., 2016). Here, we present novel variants of *SUCLG1* gene in a Chinese infant with hypotonia, psychomotor retardation, hearing loss and mild methylmalonic aciduria.

## 2 MATERIALS AND METHODS

### 2.1 Ethical compliance

This research was approved by the ethics committees of The First Affiliated Hospital of Wenzhou Medical University.

### 2.2 Participants

The proband and parents were enrolled from The First Affiliated Hospital of Wenzhou Medical University. This patient was admitted to neonate ICU (NICU) for respiratory distress and hypotonia. Written consent was obtained from parents of this patient prior to commencing the study.

### 2.3 Whole exome sequencing (WES) and Sanger sequencing

Peripheral blood samples (3–5 ml) were obtained from the proband and parents in EDTA tubes. Genomic DNA from lymphocytes was extracted, purified, and fragmented into random segments. Genomic DNA was then captured using the Agilent SureSelect Human All Exome V6 Kit (Agilent Technologies). After DNA library preparing, high‐throughput sequencing was performed using Illumina HiSeq X Ten system (Illumina, Inc.), with a reading length of 150 bp. In general, test platform examined >95% of the targeted regions with sensitivity of above 99%. The average coverage of the target bases was >150×. The exome sequencing resulted in >12 GB of clean data. Sequence alignment was performed according to the GRCh38/hg38 human reference genome sequence using BWA aligner (Burrows‐Wheeler alignment tool, version 0.7.15). GATK program was conducted to identify the insertions or deletions. Variants were annotated and filtered using TGex (https://geneyx.com/geneyxanalysis/). The main reference databases included population databases (dbSNP, 1000G, and gnomAD) and disease databases (Human Gene Mutation Database [HGMD], ClinVar, OMIM, UCSC and DECIPHER).Common variants were screened according to their frequency in the Exome Aggregation Consortium (ExAC03) database (minor allele frequency [MAF] < 0.05). The pathogenicity of the variants was classified according to the standards and guidelines of the American College of Medical Genetics and Genomics (ACMG) (Richards et al., 2015).

### 2.4 Reverse transcription and quantitative real‐time PCR (qRT‐PCR)

The relative mitochondrial DNA (mtDNA) copy number in blood leucocytes were measured by real time polymerase chain reaction (StepOne™ Real‐Time PCR System) (ThermoFisher Scientific) using complementary primers to *MT‐TL1* gene and corrected by simultaneous measurement of the nuclear DNA using complementary primers to *BM2* gene. PCR was performed in a final volume of 20 μl containing 1 μl of DNA template which was mixed with 10 μl SYBR green SuperReal Mix Plus (Tiangen, Shanghai, China), 0.5 μl of each primer (10 mM), and 8 μl H_2_O. The PCR conditions were divided into 3 steps. The first one was the hold step, 15 min at 95°C, followed by 40 cycles of denaturation at 95°C for 10 s, annealing at 60°C for 30 s and a final step of extension at 72°C 30 s followed by 1 min at 60°C. Reactions were performed in the BIO‐RAD CFX Real‐Time System (Bio‐Rad). The qRT‐PCR was replicated three times. In order to calculate the mtDNA content (mtDNA/B2M ratio) we used the formula: mtDNA content = 1/2^ΔCt^, where ΔCt = CtmtDNA−CtB2M. The primers are listed as follow: *MT‐TL1*‐forward:CACCCAAGAACAGGGTTTGT, *MT‐TL1*‐reverse: TGGCCATGGGTATGTTGTTA; *B2M*‐forward: TGCTGTCTCCATGTTTGATGTATCT, *B2M*‐reverse: TCTCTGCTCCCCACCTCTAAGT.

### 2.5 Bioinformatic analysis

The sequence alignment of the SUCLG1 protein was performed using the ClustalW program (http://bioinfo.hku.hk/services/analyseq/cgi‐bin/clustalw_in.pl). The potential effect of novel missense variant was investigated using Polyphen‐2 software (http://genetics.bwh.harvard.edu/pph2/). The assessment of the possible impact of the variant on the protein transport by predicting the mitochondrial presequence, and its cleaved position, was performed with Mito‐Fates software (http://mitf.cbrc.jp/MitoFates/cgi‐bin/top.cgi). ProtParam (http://web.expasy.org/protparam/) was used to allow the computation of various physical and chemical parameters of the studied protein. The computed parameters include the molecular weight, theoretical pI, amino acid composition, extinction coefficient, aliphatic index and grand average of hydropathicity (GRAVY). ProtScale (http://web.expasy.org/protscale/) was used for computing and representing the profile produced by amino acid scale on the protein. The three‐dimensional structure of SUCLG1 protein was conducted by SWISS_MODEL (http://swissmodel.expasy.org/).

## 3 RESULTS

### 3.1 Clinical data

A 1‐day old male newborn conceived by in vitro fertilization (IVF) was referred to hospital for respiratory distress and hypotonia. The baby boy was born by spontaneous vaginal delivery to a 28‐year‐old gravida 3 para 1 mother at the 38th week of gestation. At birth, he had 2550 g of weight, 48 cm of length and 33 cm of head circumference. The patient did not have surface anomalies and was vigorous at birth with Apgar scores of 9 at 1 min and 10 at 5 min. Maternal pregnancy was uncomplicated. Family history included a spontaneous abortion at 8 weeks of gestation and an exfetation. No consanguinity was reported. At about 30 h of life, the patient became dyspneic and mottled. Arterial blood gas (ABG) analysis showed severe metabolic acidosis (pH 7.08, bicarbonate 6.3, base excess of −22, lactate >12). A full sepsis workup was negative and the infant remained acidosis (lactate often >12) despite numerous fluid boluses and administration of sodium bicarbonate. His liver enzymes were slightly elevated (ALT 68 U/L, AST 57 U/L) (Table 1). Urine organic acid analysis by gas chromatography/mass spectrometry (GC–MS) showed that the levels of lactic acid, pyruvic acid, 3‐hydroxybutyric acid, and methylmalonic acid (MMA) were all increased. Blood ammonia level and blood tandem mass spectrometry findings were normal (Table 1).

**TABLE 1:** The results of proband detected in blood and urine

Serum	Value	References
Glutamic‐pyruvic transaminase (ALT) (U/L)	68	9–50
Glutamic‐oxalacetic transaminase (AST) (U/L)	57	9–50
Lactic acid (mmol/L)	>12	0–2.0
Ammonia (μmol/L)	60	0–100
Creatine kinase (CK)	89	58–110
Free carnitine (C0) (μmol/L)	12.36	9.50–60.0
Propionyl carnitine (C3) (μmol/L)	3.41	0.40–5.00
Succinylcarnitine (μmol/L)	0.06	0.01–0.12
Urine		
Lactic acid	256.4	0.0–13.0
Pyruvic acid	174.1	0.0–30.0
Methylmalonic acid (MMA)	13.1	0–4.0
3‐hydroxypropionic acid	12.9	0–4.0
Methylcitric acid	1.2	0–0.7

**FIGURE 1:**
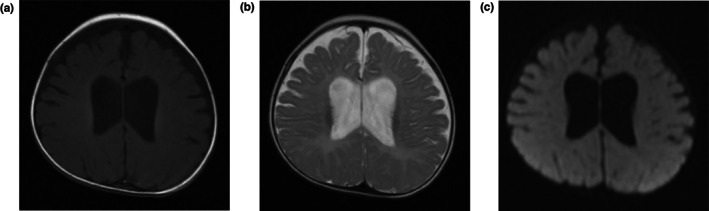
Brain MRI of the patient showed cerebral dysplasia and bilateral lateral cerebral ventriculomegaly. (a) T1W; (b) T2W; (c) DW.

During follow‐up, the patient presented with remarkable developmental delay and cognitive impairment. Audiometry revealed bilateral hearing loss. Brain magnetic resonance imaging (MRI) showed cerebral dysplasia and bilateral lateral cerebral ventriculomegaly at the age of 5‐month (Figure 1a–c). At 18 months, he could not balance head, laugh, babble, turn over or sit. Signs and symptoms of systemic involvement were also noted with thin, recurrent vomiting, gastrointestinal reflux, as well as incontinence of stool and urine. Physical examination showed muscle weakness in limbs with absent patellar and Achilles tendon reflexes. The Babinski and Chaddock signs were negative.

### 3.2 Variant detection

**FIGURE 2:**
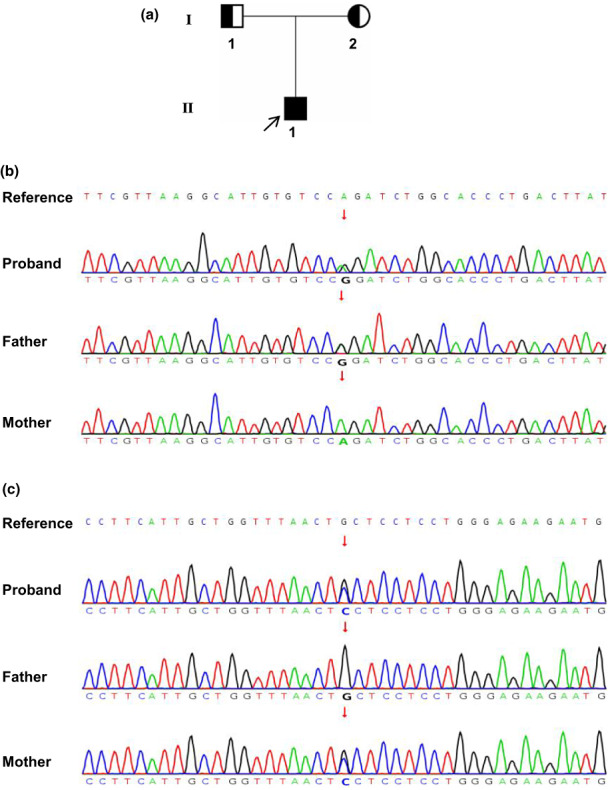
Pedigree of the studied family and sequence chromatograms showing the variants in SUCLG1 gene. (a) Pedigree of the studied family. (b) Sanger sequencing showing *SUCLG1*c.601A>G variant. (c) Sanger sequencing showing *SUCLG1*c.871G>C variant. The black arrow indicates the proband. The red arrows indicate variant sites.

Two novel compound heterozygous variants in *SUCLG*1 (c.601A>G in exon6 and c.871G>C in exon8) (NM_003849.3) were identified in the proband (II‐1) by WES (Figure 2a). The variant c.601A>G inherited from father (I‐1) substitutes conserved arginine (Arg) residue to glycine (Gly) at position 201(Figure 2b,d). The missense variant c.871G>C inherited from mother (I‐2) changed alanine (Ala) to proline (Pro) at position 291 (Figure 2c,e). These variants are rare and have not been previously presented in 1000 Genomes Consortium Phase 3 (based on GRCh38) (http://www.ensembl.org/), dbSNP (https://www.ncbi.nlm.nih.gov/SNP/), or the Exome Variant Server databases (http://evs.gs.washington.edu/EVS/).

### 3.3 Mitochondrial DNA analysis

**FIGURE 3:**
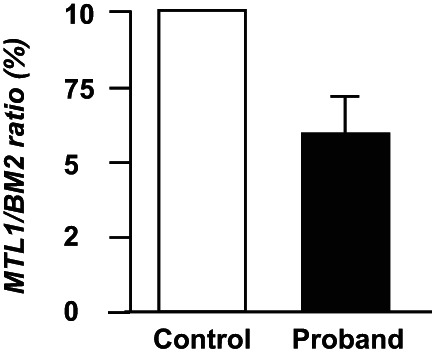
Relative mtDNA contents (MTL1/BM2) in peripheral blood of the proband and age‐matched controls. The mtDNA content of proband was normalized by mtDNA in controls.

To investigate the impacts of p.R201G and p.A291P on the mitochondrial DNA content, we detected the mtDNA levels of the conserved mitochondrial gene *MT‐TL1* from peripheral blood of the proband and healthy individuals by qPCR. Meanwhile, the nuclear genome encoded *BM2* gene was used to assess nuclear genomic DNA level. By comparison to two age‐matched normal controls, our patient had a decrease of 38.0% in *MT‐TL1/BM2* ratio (Figure 3), indicating that Arg201Gly and Ala209Pro in SUCLG1 could cause mtDNA depletion.

### 3.4 Computational analysis

#### 3.4.1 Effect on the protein transport

**FIGURE 4:**
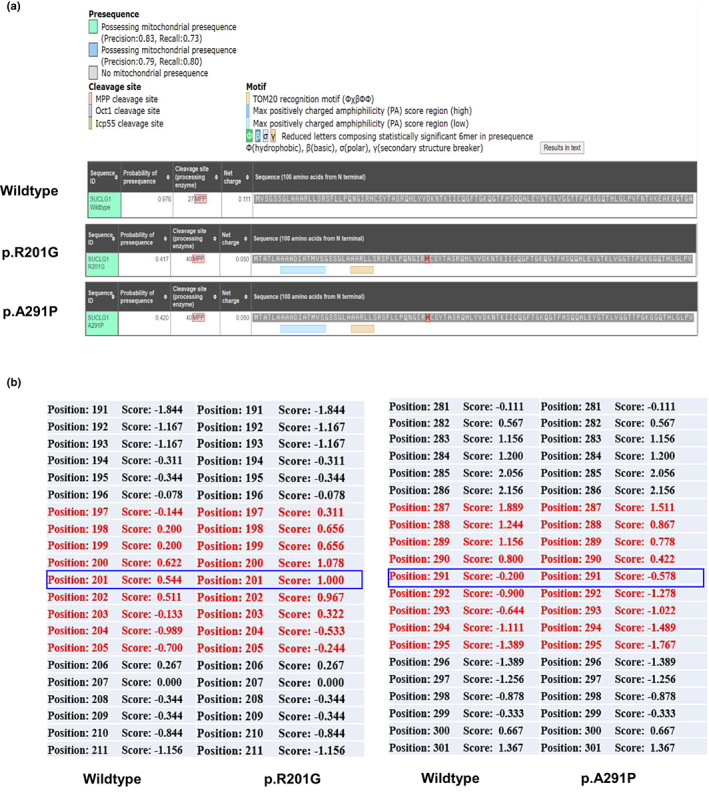
(a) Mitofates prediction in human's wild type and mutated SUCLG1 mitochondrial targeting sequences. (b) Hydrophobicity scale in SUCLG1is compared in the wildtype and mutated p.R201G and p.A291P proteins. Blue frames show changes in hydrophobicity scales.

We used MitoFates to predict SUCLG1 protein mitochondrial localization by analyze the presence of N‐terminal mitochondrial targeting signals and their cleavage sites (Fukasawa et al., 2015). The results showed that both c.601A>G(p.R201G) and c.871G>C(p.A291P) could induce the loss of mitochondrial presequence, where the scores were decreased from 0.978 to 0.417 and 0.420, respectively (Figure 4a). These data imply that these variants might affect the transportation of SUCLG1 protein from cytoplasm to mitochondrial matrix.

#### 3.4.2 Effect on structure and hydrophobicity

By Expasy proteomics, 201G SUCLG1 was predicted to have a reduced isoelectric point (pI) of 8.89 (compared to 9.01 for the wild type). The molecular weight (MW) was also slightly decreased (36.151 kDa for 201G SUCLG1 vs. 36.250 kDa for wild type). The grand average of hydropathicity (GRAVY) remarkably increased to 1.000 (compared to 0.544 for the wildtype). For 291P SUCLG1, the pI level was predicted to 9.01, which is the same to the wildtype. The MW was slightly increased to 36.276 kDa, and the GRAVY was declined to −0.578 (compared to −0.200 for the wildtype). By ProtScale analysis (Fukasawa et al., 2015), the scales of local hydrophobicity at and near the substitutions of R201G and A291P were altered (Figure 4b), which might lead to a decrease in SUCLG1 flexibility and disturb the interaction of SUCLG1 with other molecules, notably CoA.

#### 3.4.3 Effect on 3D structure

**FIGURE 5:**
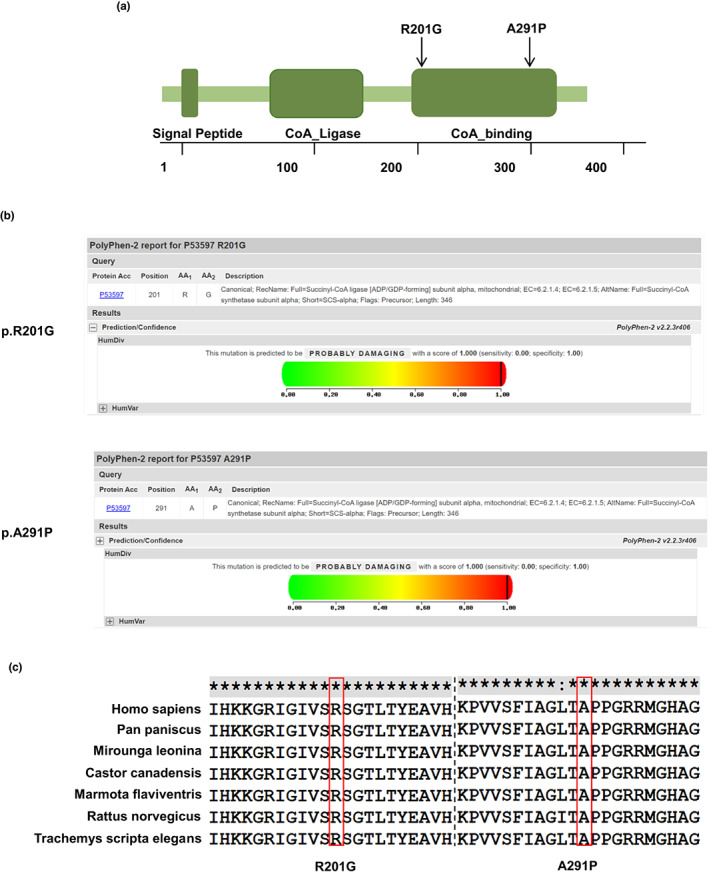
Scheme and molecular model of SUCLG1 protein. (a) Schematic view of SUCLG1 protein. Both variants reported here fall in the CoA_binding domain. (b) The scores of Polyphen‐2 Prediction of p.R201G and p.A291P on HumVar models. (c) Evolutionary conservation of the p.R201 and p.A291 from elegans to human. The positions of variants are indicated by red rectangles.

Computer‐based algorithms SIFT and PolyPhen‐2 predict p.R201G and p.A291P to be deleterious both with score of 1.000 on HumVar models (Figure 5b). Both missense variants affected residues located in a region important for binding of SUCLG1 to CoA (Figure 5a) (Molaei et al., 2020). Crucially, these two variants involve residues are highly conserved from elegans to human (Figure 5c). These data underscore that 201R and 291A are key to normal biological function of SUCLG1 protein.

**FIGURE 6:**
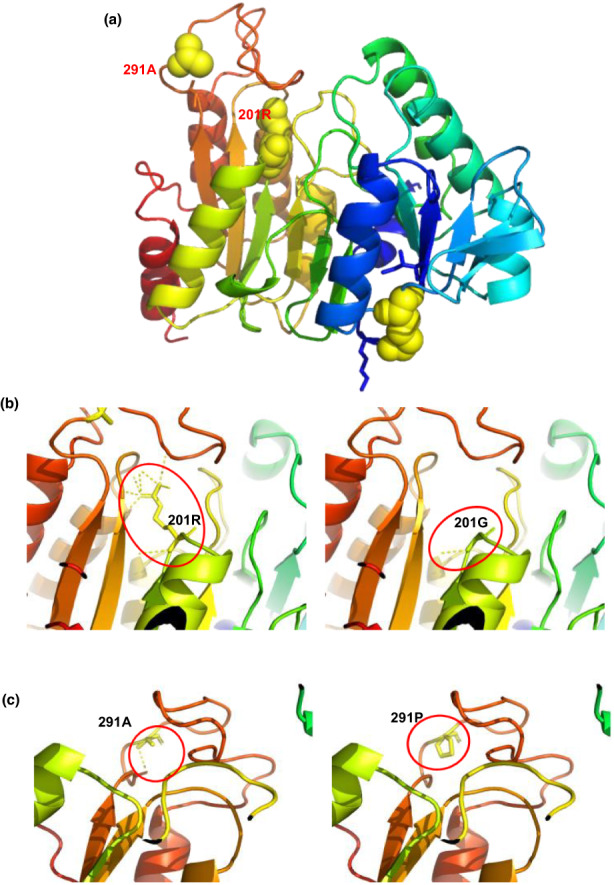
Modeled structure of the human SUCLG1 protein. (a) Structure of the wildtype SUCLG1 protein. 201R and 291A residues are annotated in red font. (b,c) The hydrogen bonding network is illustrated by the yellow dashed lines surrounding R201 and A291. (b) 201R is involved in hydrogen bonds with the residues 255I, 257‐259EIG and 301G, while the 201G variation removes all the hydrogen bonds except 255I. (c) 291A may create an ionic bound with 288A, while the mutant 291P could potentially abolish that only one hydrogen bond.

To investigate the eventual effects of the non‐synonymous variations on changing p.R201G and p.A291P of SUCLG1 protein, we modeled and compared above variants. 3D Model revealed that 201R involved in hydrogen bonds with the residues 255I, 257‐259EIG and 301G, while the 201G variation removes all the hydrogen bonds except 255I (Figure 6a,b). In addition, the missense variant Ala291Pro could potentially abolish the only one hydrogen bond with Ala at 288‐position (Figure 6a,c). Based on these observations, such variants might result in the possible inability to bind the water molecule, which is crucial in the ligation reaction.

## 4 DISCUSSION

*SUCLG1*‐related encephalomyopathy is an autosomal recessive disorder. Case with MTDPS9 are rare and about 30 SUCLG1 variants have been reported, including missense, splice site and nonsense variants (Rouzier et al., 2010; Van Hove et al., 2010; Carrozzo et al., 2016; Liu et al., 2016; Molaei et al., 2020; Bychkov et al., 2021). Here, we present our findings in a male infant with mitochondrial encephalomyopathy, which could be attributable to compound heterozygous variants in the *SUCLG*1 gene. WES found novel compound *SUCLG1* c.601A>G (p.R201G) and c.871G>C (p.A291P) variants, inheriting from normal father and mother, respectively. SUCLG1 p.201Arg and p.291Ala were highly conserved residues during evolution from elegans to human, that implies variants in these positions would possibly affect function. Then computational analysis predicted these missense variants might alter SUCLG1 structure stability, mitochondrial translocation and hydrogen bonds. Functional studies proved that patient cells had mtDNA depletion and supported pathogenicity of *SUCLG1* c.601A>G and c.871G>C. Thus, our investigations suggest that *SUCLG1* c.601A>G and c.871G>C variants may be disease‐causing in the family.

The typical manifestation of MTDPS9 is normal pregnancy usually followed by onset of hypotonia in early postnatal life. Failure to thrive and feeding difficulties often need tube‐feeding, and infants develop muscular atrophy, cognitive impairment and growth retardation with overt reduction of height and weight. About 20%–50% patients present sensorineural hearing impairment. But epilepsy is rare. The disorder is also associated with a shortened life span. Brain MRI may show bilateral basal ganglia hyperintensities (80%), cerebral atrophy (30%) and leukoencephalopathy (20%) (Van Hove et al., 2010; Landsverk et al., 2014; Donti et al., 2016; Liu et al., 2016; Chinopoulos et al., 2019). Analysis of urine usually finds mild increase of methylmalonic acid. In this study, the male neonate manifested with hypotonia and lactic acidosis on the second day after birth. His urine MMA level was about 3‐fold higher than normal reference. At 18‐month, he could not raise head, laugh, babble, and turn over, suggesting severe psychomotor retardation. MRI showed cerebral dysplasia and audiometry revealed bilateral hearing loss. Thus, the clinical presentations and lab investigations were consistent with the features of MTDPS9.

The pathogenesis of the MTDPS9 remains to be discovered. Since mitochondrial is a major source of energy for a majority of cellular processes, it is not surprising that paucity in mitochondrial function may induce developmental malformations. Sufficient mtDNA copy number is essential for mitochondrial respiratory chain reaction and energy biosynthesis. SCS forms a complex with the mitochondrial nucleoside diphosphate kinase (NDPK), which functions to salvage of deoxyribonucleotides by catalyzing the reversible transfer of a terminal phosphoryl group between di‐ and tri‐phosphonucleosides (Kowluru et al., 2002; El‐Hattab and Scaglia, 2013). An absence of this complex formation in SCS deficiency has been suggested to disturb the kinase activity, resulting in decreased mtDNA synthesis leading to mtDNA depletion (Kowluru et al., 2002). Usually, a reduced content of mtDNA (typically 15%–50%) may be found in tissues or peripheral blood leucocytes (Landsverk et al., 2014; Molaei et al., 2020). Likewise, we also observed that mtDNA content in the peripheral leukocytes of proband was about three times lower than in normal controls, which indicates that c.601A>G and c.871G>C variants may prevent SUCLG1 function. Above results could interpret the phenotype of mitochondrial DNA depletion syndrome in the proband. According to the ACMG guidelines, variants c.601A>G and c.871G>C in *SUCLG1* are classified as variants of unknown significance (VUS) (Richards et al., 2015). However, based on our bioinformatic analysis and mtDNA results, these variants are prone to be deleterious and could be reclassified as probably affecting function. The underlying mechanisms by which c.601A>G and c.871G>C variants weaken the function of SUCLG1 protein need further studies.

In summary, we identified the compound heterozygous variants c.601A>G (p.R201G) and c.871G>C (p.A291P) in *SUCLG1*, which may cause MTDPS9 in this Chinese infant. Our study expands the spectrum of *SUCLG1* variants involved in mitochondrial DNA depletion syndrome. For infants with severe psychomotor retardation and mild methylmalonic aciduria, molecular genetic analysis is critical for diagnosis and clinical management.

## AUTHOR CONTRIBUTIONS

Conceptualization: Dan Wang. Data collection: Wei Chen, Yue Xu, Chao‐sheng Lu, and Mian‐mian Zhu. Investigation: Yi‐ming Chen and Rong‐yue Sun. Bioinformatic analysis: Yi‐ming Chen and Yihong Wang. Methodology: Rong‐yue Sun, Yihong Wang, Yuan Chen and Jiaming Shi. Supervision: Dan Wang. Writing‐original draft: Yi‐ming Chen and Wei Chen. Writing‐review and editing: Yi‐ming Chen and Dan Wang.

## CONFLICT OF INTEREST

The authors have no conflicts of interest to declare.
